# Maillard Reaction-Derived Carbon Nanodots: Food-Origin Nanomaterials with Emerging Functional and Biomedical Potential

**DOI:** 10.3390/pharmaceutics17081050

**Published:** 2025-08-13

**Authors:** Gréta Törős, József Prokisch

**Affiliations:** 1 Institute of Animal Science, Biotechnology and Nature Conservation, Faculty of Agricultural and Food Sciences and Environmental Management, University of Debrecen, Böszörményi Street 138, 4032 Debrecen, Hungary; jprokisch@agr.unideb.hu; 2 Doctoral School of Animal Husbandry, Faculty of Agricultural and Food Sciences and Environmental Management, University of Debrecen, Böszörményi Street 138, 4032 Debrecen, Hungary

**Keywords:** maillard reaction, carbon nanodots, food processing, nanomaterials, drug delivery, melanoidins, advanced glycation end products, bioactive compounds, antioxidants, heat-induced reactions

## Abstract

The Maillard reaction (MR), a non-enzymatic interaction between reducing sugars and amino compounds, plays a pivotal role in developing the flavor, color, and aroma of thermally processed foods. Beyond its culinary relevance, the MR gives rise to a structurally diverse array of compounds, including a novel class of fluorescent nanomaterials known as carbon nanodots (CNDs). These Maillard-derived CNDs, although primarily incidental in food systems, exhibit physicochemical characteristics—such as aqueous solubility, biocompatibility, and tunable fluorescence—that are similar to engineered CNDs currently explored in biomedical fields. While CNDs synthesized through hydrothermal or pyrolytic methods are well-documented for drug delivery and imaging applications, no studies to date have demonstrated the use of Maillard-derived CNDs specifically in drug delivery. This review examines the chemistry of the Maillard reaction, the formation mechanisms and characteristics of food-based CNDs, and their potential functional applications in food safety, bioactivity, and future biomedical use. Additionally, it critically evaluates the health implications of Maillard reaction products (MRPs), including both beneficial antioxidants and harmful by-products such as advanced glycation end-products (AGEs). This integrated perspective highlights the dual role of MR in food quality and human health, while identifying key research gaps needed to harness the full potential of food-origin nanomaterials.

## 1. Introduction

The Maillard reaction is a well-known chemical process in food science because it affects cooked foods’ flavor, color, and nutrition. First described by French chemist Louis Maillard in 1912, it involves a complex reaction between sugars and amino groups from amino acids, peptides, or proteins. This reaction happens during cooking methods like baking, roasting, frying, and grilling. Heat helps sugars react with amino groups, forming early compounds (called Amadori products) and many other substances that influence food quality [1].

The MR is of paramount importance for the sensory qualities of foods, as it produces the characteristic brown pigments known as melanoidins and generates a broad spectrum of volatile and non-volatile flavor compounds that define the aroma and taste of numerous culinary staples such as bread crust, roasted coffee, grilled meat, and caramelized vegetables [2,3]. These sensory attributes arise from forming melanoidins (brown pigments) and a broad spectrum of Maillard reaction products (MRPs), including heterocyclic compounds like pyrazines, furans, thiophenes, and pyrroles. Many of these compounds contribute richly to flavor and exhibit antioxidant activity, enhancing food stability and offering potential health benefits [4,5].

Despite its prevalence in cooking and food manufacturing, the Maillard reaction is a highly complex chemical process that involves numerous intermediates and multiple reaction pathways. Its progression and the nature of its products are strongly influenced by various factors, including temperature, pH, moisture content, the type and concentration of reactants, and water activity—a measure of the availability of unbound (free) water in a system that can participate in chemical reactions [6]. These parameters interact in intricate ways, making it challenging to predict or control the reaction’s outcome without careful optimization [7].

Early studies in this field often used simplified model systems of single sugars and amino acids to understand basic reaction mechanisms. However, real food matrices introduce many variables—including complex proteins, peptides, lipids, and polysaccharides—which complicate the prediction and control of MR behavior in practical applications [8,9].

Beyond flavor and color development, the MR is also responsible for forming advanced glycation end products (AGEs), a class of late-stage compounds with implications in human health. Dietary intake of AGEs has been linked to various chronic conditions, including diabetes, cardiovascular diseases, and neurodegenerative disorders [5,10]. As a result, current research focuses on achieving a balance between the desirable sensory qualities derived from the MR and the mitigation of potential health risks associated with its by-products.

Recent advancements in analytical techniques—such as high-resolution mass spectrometry, nuclear magnetic resonance (NMR), and gas chromatography-mass spectrometry (GC-MS)—have provided more profound insights into the molecular complexity of MRPs [11,12]. These developments have also fueled interest in novel applications of the Maillard reaction, such as the development of natural antioxidants, flavor enhancers, and carbon-based nanomaterials derived from thermally processed food components [13,14].

**Figure 1:**
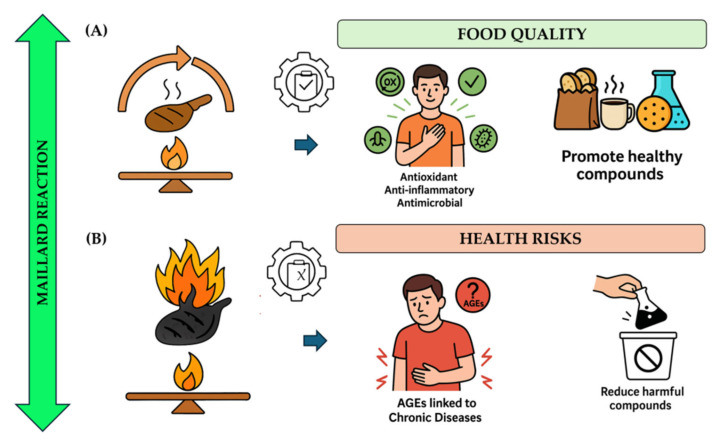
Impact of Maillard Reaction Control on (**A**) Food Quality (e.g., flavor, color, antioxidant activity) and (**B**) Health Risk (e.g., formation of AGEs, acrylamide).

The goal of this review is to provide a balanced perspective that highlights innovative approaches for harnessing the functional benefits of Maillard reaction products, especially carbon nanodots, in food and biomedical applications, while also addressing strategies to minimize associated health risks. Figure 1 shows a schematic overview illustrating the impact of Maillard reaction control on food quality and health: (A) highlights the positive effects of MRPs, including enhanced flavor, color, and antioxidant activity; (B) depicts the associated health risks, such as the formation of acrylamide, advanced glycation end-products (AGEs), and other potentially toxic compounds due to uncontrolled or excessive Maillard reactions.

## 2. Methodology of the Review

A comprehensive and reproducible literature review was conducted using systematic searches across academic databases, including ScienceDirect, SpringerLink, PubMed, and Google Scholar. Keyword combinations including “food processing,” “melanoidins,” “flavor compounds,” “antioxidants,” “bioactive compounds,” “dietary exposure,” “heat-induced reactions,” “carbon nanodots,” and “nanomaterials” were employed. The review prioritized studies published between 2019 and 2024 to capture the most recent advancements, while also incorporating highly cited earlier works. Only peer-reviewed original research and review articles written in English were included. The selected studies examined Maillard reaction products (MRPs) within food systems, emphasizing their safety, processing optimization, biological impacts, and their emerging roles in nanomedicine, particularly regarding carbon nanodots (CNDs) as drug delivery agents. Studies were excluded if full texts were unavailable, if they were limited to conference abstracts, or if they lacked sufficient methodological detail. Additional selection criteria included journal impact factor, author expertise, and relevance to the research question. Articles were first screened by title and abstract, followed by full-text assessment for final inclusion. Key findings were synthesized and organized using tables and figures to provide a clear and integrative overview.

## 3. Understanding the Maillard Reaction (MR)

### 3.1. The Stages of MR

The Maillard Reaction (MR) is a non-enzymatic chemical process that occurs when foods containing proteins, peptides, and carbohydrates are exposed to heat. It initiates with a reducing sugar reacting with amino acids such as lysine or arginine, leading to the formation of early glycation products [15,16]. These initial products may transform into reactive intermediates with α-dicarbonyl structures. Unlike enzyme-driven reactions, the MR is a non-enzymatic process involving sugars, amino acids, thiol compounds, and polyphenols [9].

The MR is critical in food science due to its contribution to flavor, aroma, browning, and texture. It is active in nearly all thermally processed or stored foods, especially those rich in proteins and sugars [17]. However, it also poses challenges outside food systems—for instance, in high-temperature industrial environments where it can cause surface fouling [18,19].

**Figure 2:**
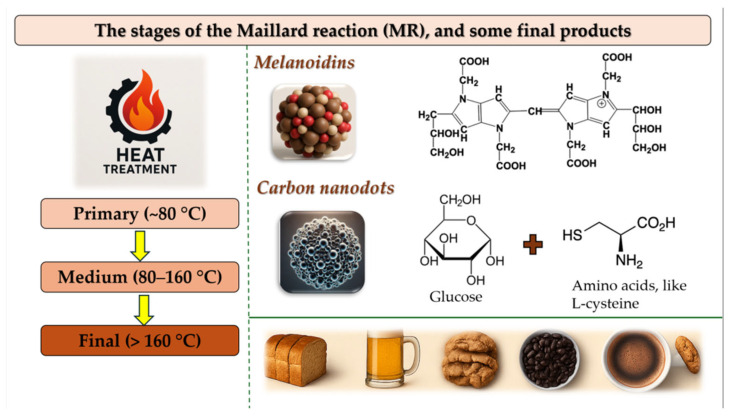
The main stages and products of MR.

Figure 2 shows a schematic overview of the three stages of the Maillard Reaction. Early stage: formation of Amadori products; intermediate stage: Strecker degradation and flavor compound formation; final stage: production of high molecular weight melanoidins and carbon-rich nanostructures (CNDs).

Early glycation reactions dominate in the initial stage, typically occurring within the temperature range of room temperature up to ~80 °C. However, it is essential to note that reaction rates increase significantly with temperature, while Schiff base formation and Amadori rearrangement can occur slowly at ambient temperatures (20–30 °C), these reactions proceed much more rapidly as temperatures approach 60–80 °C [20]. In this stage, reducing sugars interact with amino acids to form unstable Schiff bases, which subsequently rearrange into more stable intermediates such as glycosylamines and Amadori products. Representative early-stage compounds—such as Gly-Amadori, Cys-Amadori, and TTCA (2,4,6-trihydroxy-5-(2-hydroxyethyl)-3-pyridinecarboxylic acid)—serve as browning markers and are closely associated with the onset of flavor development [21].

As temperatures increase into the intermediate range (80–160 °C), the reaction complexity escalates. This middle phase is characterized by the formation of various reactive intermediates through dehydration, fragmentation, and Strecker degradation. Notably, Strecker degradation contributes significantly to flavor chemistry by converting amino acids into aldehydes, ketones, and pyrazines—compounds responsible for savory, roasted, and nutty aroma profiles [22].

The final stage, occurring at temperatures above 160 °C, involves high-energy transformations such as polymerization, cyclization, and condensation reactions. These processes generate melanoidin, high molecular weight, nitrogen-rich polymers responsible for the deep brown color of cooked foods, and a wide range of volatile and non-volatile compounds that significantly influence sensory attributes [23].

The MR is the primary driver behind developing complex flavors and browning in cooked foods [8,24]. MRPs formed during the intermediate and final stages are responsible for iconic aromas such as caramel, toasted nuts, and umami-rich notes [3,25]. Chemically, these include a broad spectrum of volatile compounds, notably nitrogen—and carbon-containing molecules such as pyrazines, furans, and thiophenes [26].

Given their profound sensory impact and general safety, MRPs are a significant focus in food science research. Modern studies aim to optimize Maillard reaction conditions to enhance desired sensory characteristics while mitigating adverse outcomes such as the formation of off-flavors, nutrient degradation, and potentially allergenic or toxic compounds [21,22,23]. This balance between flavor enhancement and food safety remains a central challenge in developing high-quality, thermally processed food products.

### 3.2. Factors Influencing the Maillard Reaction in Food Processing

The Maillard reaction (MR) is central to food processing, creating beneficial and harmful compounds. While it enhances flavor, aroma, and color, it can also lead to unwanted browning, off-flavors, and health risks, particularly by forming potentially toxic compounds like acrylamide and furan. These Maillard Reaction Products (MRPs) are mostly bound within food matrices, complicating their nutritional relevance and bioavailability.

Despite concerns, some MRPs offer health benefits such as antioxidant and anti-inflammatory effects [11,27]. However, the mechanisms behind these effects and their absorption during digestion remain unclear. As MR progresses, especially in protein-rich foods, it can compromise visual appeal and nutritional value. It is essential to regulate the reaction by controlling interactions between amino acids and reducing sugars [6,8].

Given the complexity of the MR, which produces thousands of different compounds, advanced tools like gas chromatography–mass spectrometry (GC-MS) are vital for analyzing its products [28,29]. Studies emphasize that MR conditions must be optimized to balance the production of desirable compounds (like flavor enhancers and antioxidants) with minimizing harmful byproducts (such as heterocyclic amines and acrylamide) [30,31].

**Figure 3:**
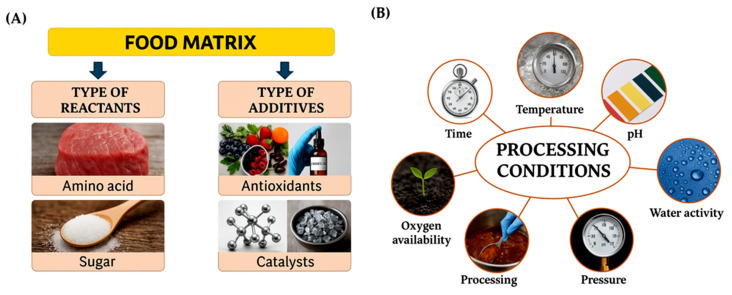
The summarization of the factors affecting MR, including the (**A**) whole food matrix and (**B**) processing conditions.

As shown in Figure 3, the rate and outcome of the Maillard reaction are influenced by a range of intrinsic and extrinsic factors. Intrinsic factors include the type and concentration of reactants (such as specific amino acids and reducing sugars), the pH, which affects the reactivity and stability of intermediates, and the water activity (aw), which refers to the availability of unbound water in the system and plays a key role in facilitating or limiting reaction progress.

## 4. Types of Maillard Reaction Products

MR is key in developing foods’ color, flavor, and aroma. MRPs can be broadly divided into flavor and color compounds, significantly affecting food’s sensory and chemical properties [6]. In addition, carbon nanoparticles, along with their role in the delivery of bioactive compounds and the digestive system, can also form during this process [32]. Understanding how these compounds are formed, and their roles is essential for improving food processing, product quality, and safety.

### 4.1. Flavor Compounds

The Maillard reaction generates volatile and semi-volatile flavor compounds crucial in processed foods’ aroma and taste profiles. These compounds arise primarily from complex interactions between reducing sugars and amino acids, further transforming through Strecker degradation, condensation, and cyclization reactions [8,33].

Table 1 presents some examples of key Maillard-derived flavor active compounds, such as acids, alcohols [34], aldehydes [35], ketones [36], carbonyls [37], sulfur-containing compounds [38], and heterocyclic structures [34]. MR-formed flavor compounds contribute to meaty, roasted, nutty, and caramel-like notes, which are highly desirable in food products [39].

### 4.2. Color Compounds

In addition to flavor, the Maillard reaction is also responsible for forming color compounds that contribute to the brown appearance of thermally processed foods. These browning products are typically high-molecular-weight, nitrogen-containing polymers and aromatic compounds [42].

The development of these pigments—collectively known as melanoidins—occurs during the later stages of the Maillard reaction, particularly under acidic or neutral pH conditions. These compounds affect food aesthetics and may possess antioxidant, antimicrobial, and metal-chelating properties [43]. Conversely, some MR colorants, such as 5-hydroxymethylfurfural (HMF), are considered potential process contaminants and require monitoring for food safety [44]. Table 2 provides an overview of typical Maillard-derived color compounds.

### 4.3. Recent Findings on the Formation of Carbon Nanodots (CNDs) Through the Maillard Reaction

Carbon nanodots (CNDs) can form as incidental by-products during the Maillard reaction (MR), particularly when nitrogen-rich and carbohydrate-rich compounds undergo heat-induced transformations. These food-derived nanomaterials typically exhibit small particle sizes (<10 nm), strong fluorescence, and surface functionalities such as hydroxyl, carboxyl, and amino groups. Their photoluminescence is highly dependent on reaction temperature, pH, and precursor composition [51,52].

While synthetic CNDs—produced through controlled pyrolytic or hydrothermal techniques—have been extensively studied for use in drug delivery, bioimaging, and biosensing [53], no published studies have yet demonstrated the use of Maillard-derived CNDs for drug delivery applications. However, their structural similarity to engineered CNDs suggests they may serve as promising candidates in the future.

Recent reports have identified Maillard-derived CNDs in food matrices such as coffee brews [52], oyster mushroom powder [51], and baked goods [54], with studies confirming their antioxidant activity, fluorescence, and low cytotoxicity in HepG2 cells [55]. Importantly, these CNDs are not synthesized with biomedical use in mind and are present at low, uncontrolled concentrations in food. As such, they currently play functional roles in food quality monitoring and potential bioactivity rather than targeted therapeutic delivery [56].

Further research is needed to isolate Maillard-derived CNDs in sufficient purity and yield, assess their toxicological profiles, and evaluate their capacity for functionalization and cargo delivery.

Table 3 summarizes the key findings from recent studies linking Maillard conditions and CND formation. This growing body of work highlights an exciting research frontier: food-origin nanodots as bioactive or diagnostic materials—though their biomedical deployment remains an unrealized potential.

**Figure 4:**
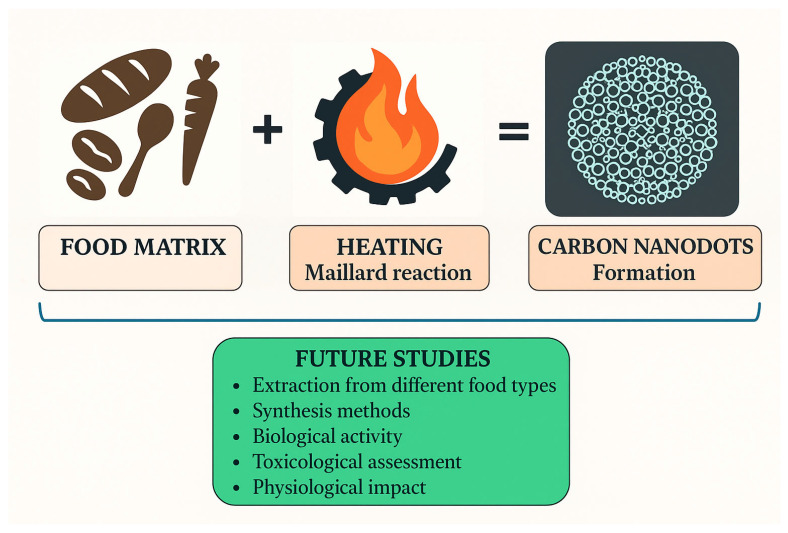
A novel compound formed through the Maillard reaction (MR) and some future aspects.

Our findings conclude that future efforts should focus on extracting pure carbon nanodots from heat-treated food products and comprehensively evaluating their biological activity, including toxicity assessment and physiological impact, to assess their potential efficacy fully, as summarized in Figure 4.

These nanoparticles possess intriguing optical and bioactive properties, such as fluorescence and immune response modulation [59,60,61]. Critical synthesis parameters such as precursor composition, thermal processing temperature, pH, and reaction time significantly influence the quantum yield, size distribution, and bioactivity of CNDs. For example, alkaline conditions enhance carbonization, while nitrogen-rich precursors (e.g., amino acids in mushrooms) facilitate nitrogen doping, which improves optical and catalytic functions [51,52].

While drug delivery applications of carbon nanodots (CNDs) are well-documented, including tunable fluorescence and targeted delivery [55,62,63,64], there are no published studies to date demonstrating the use of Maillard-derived CNDs specifically for drug delivery. Nevertheless, their observed properties—such as high biocompatibility, small size, and surface functionality—suggest they may be suitable candidates for such applications, pending future validation.

Their nano-scale dimensions and modifiable surface chemistry allow CNDs to be conjugated with therapeutic agents and targeting ligands [65], which enables them to deliver drugs to specific tissues or cellular environments, minimizing off-target effects and enhancing therapeutic efficacy [66].

In summary, CNDs bridge the gap between food chemistry and nanotechnology, as their small size, surface functionalities, and intrinsic fluorescence grant them broad applicability:

- In food science, CNDs could act as natural colorants, biosensors, or antioxidant carriers, providing functional benefits while enabling traceability and freshness indicators in packaging [67].
- In biomedicine, their biocompatibility and ability to cross cell membranes support drug delivery, tumor imaging, and bio-sensing applications [68].
- In environmental science, food-derived CNDs can have promise in pollutant adsorption, heavy metal chelation, and green catalysis [69].

## 5. Maillard Reaction in Different Food Types

Current research gaps include the influence of composition and processing on MRPs’ formation and degradation, their role in extending shelf life, and their enhancement of food products’ nutritional and sensory qualities [70,71]. Several studies have explored how MRPs function across different food products and how their properties can be manipulated to enhance their quality, as shown in Table 4.

## 6. Health Implications and Consumer Perception

Consumers typically perceive Maillard Reaction Products (MRPs) in a favorable light, mainly because they enhance key sensory attributes—such as flavor, aroma, and color—in popular foods like bread crusts, roasted coffee, grilled meats, and baked goods [76]. These sensory cues evoke familiarity, satisfaction, and indulgence, contributing to the positive image of MRPs in everyday diets. However, this widespread appreciation is not accompanied by an equivalent understanding of their potential health implications.

Most consumers are unaware that certain MRPs, including acrylamide and 5-hydroxymethylfurfural (HMF), are associated with toxicological and carcinogenic risks [44,77]. This lack of awareness stems partly from limited public education and the complex nature of food chemistry, which makes it difficult for non-specialists to connect cooking methods with long-term health outcomes. While the scientific community continues to explore both the beneficial and adverse effects of MRPs [78].

Consumer behavior is heavily influenced by taste, convenience, and cultural familiarity, which can overshadow health considerations, especially when the risks are not widely publicized or easily perceived [8]. For example, high-temperature cooking techniques such as frying and roasting are deeply embedded in culinary traditions and preferred for flavor enhancement despite their role in elevating harmful MRP levels [79].

### 6.1. Positive Effects

Despite some known risks, MRPs possess notable health-promoting properties [71]. They exhibit potent antioxidant and anti-inflammatory actions [80]. MRPs effectively scavenge harmful radicals (hydroxyl, DPPH, superoxide) [81], suppress pro-inflammatory cytokines (TNF-α, IL-1β, IL-6) in macrophages [82]. In addition, MRP-derived peptides contribute to obesity management by enhancing lipolysis and reducing appetite and fat accumulation [83,84]. These combined effects underline the potential of MRPs in the prevention or mitigation of chronic diseases such as Alzheimer’s, type 2 diabetes, and cardiovascular conditions [85].

### 6.2. Negative Effects on Health and Awareness

Some MRPs exhibit neurotoxic effects by disrupting mitochondrial function in neurons, leading to apoptosis marked by Tau protein phosphorylation, cytochrome C release [86], and caspase-3 activation [87]. They can also impair pancreatic function by inhibiting amylase secretion and damage intestinal epithelial cells, triggering gut inflammation [88].

Advanced glycation end-products (AGEs), a notable subset of MRPs, are strongly linked to chronic conditions including type 2 diabetes, polycystic ovary syndrome, cardiovascular disease, kidney failure, and Alzheimer’s disease [89]. Particularly concerning is their presence in formula milk, which has been associated with food allergies, systemic inflammation, and developmental challenges in infants [90].

Despite their widespread presence in modern diets—especially in fried snacks, baked goods, and processed meats—public awareness of the health risks posed by AGEs remains limited [71,91]. Unlike trans fats, which have been the focus of extensive public health campaigns, AGEs are still largely overlooked, even though high-temperature cooking methods significantly elevate their levels in food [92].

Promoting healthier cooking practices, such as low-to-moderate temperature techniques and non-thermal food processing, is crucial to mitigating these risks [93]. Educational efforts, similar to those used to reduce trans fat consumption, should be supported by practical and affordable dietary alternatives [94]. However, successful change must also consider consumer preferences for taste and convenience [8]. Encouraging the food industry to adopt AGE-reducing technologies and offer accessible alternatives could substantially lower dietary AGE intake and its associated health impacts [95].

### 6.3. Potential Carcinogens

Certain MRPs have been identified as probable or possible human carcinogens. For example, acrylamide is formed during high-temperature cooking of carbohydrate-rich foods such as fries, bread, and cookies. Its intake has been linked to an increased risk of cancer, and despite industry efforts to reduce its presence, consumption remains high [96].

Similarly, Heterocyclic Aromatic Amines (HAAs) are produced when meat is grilled or fried. Their formation is influenced by factors such as the type of meat, its pH, and the duration of cooking. While less extensively studied, compounds like 5-hydroxymethylfurfural (HMF)—formed during the Maillard reaction or caramelization—have been associated with tumor development in animal studies. Natural dietary compounds, such as polyphenols and sulfur-rich vegetables like garlic and onion, have been shown to reduce the formation of HAAs and other harmful MRPs [97].

Additionally, pyrazines and furan derivatives are formed during heat-induced browning processes, including drying, frying, roasting, and baking. In vitro studies, such as the Ames assay, have demonstrated that some compounds possess mutagenic or clastogenic activity so DNA can be potentially damaged [98].

## 7. Fluorescent and Functional Characteristics of CND

As summarized in Table 5, recent studies highlight the promising role of carbon nanodots (CNDs) in drug delivery systems, emphasizing their tunable fluorescence, low toxicity, and functional versatility. While various synthesis methods and biomedical applications have been explored, the need for consistent toxicological evaluation and optimization of delivery mechanisms remains essential for the safe and effective use of CNDs in therapeutic contexts [99,100,101,102,103].

## 8. General Suggestions and Future Research Directions

Maillard reaction products (MRPs), influence food color, aroma, taste, antioxidant capacity, and potentially, bioactivity [43]. Due to the diversity of MRPs, it is essential to classify and understand them in terms of molecular weight (MW). Low-molecular-weight MRPs often contribute positively to flavor and aroma, while high-molecular-weight compounds, such as melanoidins, are more associated with color formation and extending complex biological activity [104]. Carbon nanodots formed through the last stages of MR can have low and high molecular weights. Their molecular weight depends mainly on the type of precursors and the reaction conditions, which influence their optical and functional properties. For instance, higher molecular weight nanodots often exhibit more complex structures and broader biological interactions, while lower molecular weight variants tend to have more defined fluorescence features [105]. So, the structure and size of these MRPs determine their roles in food systems [106], thus encouraging researchers to fractionate them and analyze their specific impacts on food safety, functionality, and health.

Several intrinsic and extrinsic factors influence the formation and final composition of MRPs. These include pH, temperature, water activity, time, and the types of amino acids and sugars involved [76]. For example, model systems often use cysteine (a sulfur-containing amino acid) [107] and xylose (a pentose sugar) because of their high reactivity, especially in noodle processing [108]. The progress of MRs can be tracked by analyzing the extent of browning, antioxidant activity, and flavor formation at different heating stages [8].

However, despite frequent reference to these influencing parameters throughout the literature, a clear consensus is still lacking regarding their optimal ranges or threshold values for safe and functional product design [109]. For example, Maillard reactions typically accelerate above 120 °C and under low-moisture conditions; yet, such environments may also favor the formation of undesirable by-products, such as acrylamide or HMF [110]. Achieving a balance between beneficial (e.g., flavor, color, antioxidants) and harmful (e.g., AGEs, toxicants) MRPs remains a persistent challenge. Therefore, there is a pressing need for a deeper mechanistic understanding and standardization of processing variables—such as the ideal pH range (commonly 6.0–8.0), heating time, or moisture levels—to tailor MR outcomes toward safe, high-quality food and bioactive formulations [71].

A crucial aspect of current research is the effort to control and optimize MR conditions, favoring the formation of beneficial compounds while limiting the generation of harmful ones [111]. For example, researchers aim to enhance antioxidant-rich production and suppress potentially toxic MRPs [106,112]. Understanding these mechanisms is vital for designing safer, healthier processed foods.

MRPs are a double-edged sword when it comes to health. On one hand, certain MRPs exhibit antioxidant, anti-inflammatory, or antimicrobial properties [113]. On the other hand, some advanced glycation end-products (AGEs) are implicated in serious health issues [114]. AGEs, a subgroup of late-stage MRPs, are formed through repeated carbonyl-amine interactions and accumulate in protein-rich foods subjected to intense heat (e.g., grilling, baking, frying) [115].

Multiple studies have linked dietary AGEs to chronic conditions such as type 2 diabetes, cardiovascular diseases, neurodegeneration, and cancer [91]. These compounds contribute to oxidative stress and systemic inflammation through receptor-mediated pathways. They can also impair cellular functions, so it is worth following dietary guidelines and processing methods that minimize AGE formation without compromising food quality [116].

MR has been harnessed to develop new ingredients and enhance the functional properties of food products [106]. Freeze-dried MRPs, for example, have been isolated and characterized for their antioxidant and metal-chelating activities [117]. In cysteine and xylose systems, MRPs show promise in improving the umami and meaty notes in plant-based products, making them suitable for flavor enhancement in meat substitutes [118].

Moreover, certain MRPs show strong potential in modulating bioactivity, offering avenues for designing functional foods [119]. The early-stage Amadori products also serve as markers for food processing degree and help in quality control [120].

In light of increasing environmental concerns and resource limitations, the sustainability of MR-based food processes has gained momentum. One innovative approach involves converting food processing by-products—traditionally considered waste—into value-added materials like carbon nanodots (CNDs). This fits well within green chemistry and circular economy goals by reducing waste and developing eco-friendly applications [121,122,123].

The intersection of MR and nanotechnology is a fascinating frontier. Carbon nanodots (CNDs), which can be synthesized under Maillard conditions from organic matter, exhibit remarkable properties such as fluorescence, high surface area, and biocompatibility. They are being explored for various applications, from biological imaging to innovative food packaging and quality sensors [60,61,124].

**Figure 5:**
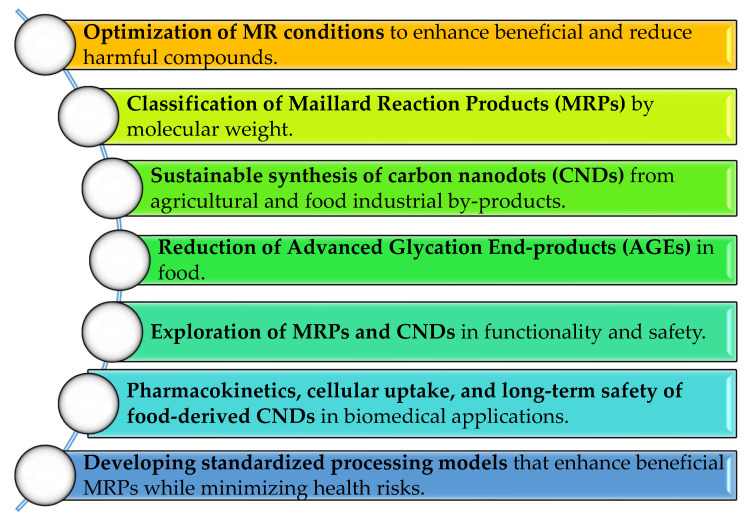
The key steps of the future research and development (R&D).

Future research will likely focus on understanding how specific MR conditions influence the formation and stabilization of CNDs, particularly those derived from aromatic compounds and heterocycles [125,126]. Expanding the range of biowaste materials used for CND synthesis could also pave the way for low-cost, sustainable technologies in food safety monitoring and biomedical applications [127,128]. Figure 5 summarizes the key concepts with several advantages for the future.

Comprehensive studies should assess the pharmacokinetics, cellular uptake mechanisms, and long-term biocompatibility of food-derived carbon nanodots (CNDs) [129], ensuring their safe integration into biomedical applications such as drug delivery and diagnostics.

Standardized processing frameworks should be established to optimize the formation of health-promoting Maillard reaction products (MRPs) while suppressing the generation of potentially toxic compounds [26].

Advancing the integration of MRPs with innovative packaging technologies could enable the development of intelligent systems capable of detecting changes in freshness, spoilage, or microbial contamination in several food systems [130].

## 9. Conclusions

The Maillard reaction plays a key role in food quality, producing both desirable flavor and color compounds and potentially harmful by-products such as advanced glycation end-products (AGEs). Recent studies have identified the incidental formation of carbon nanodots (CNDs) during Maillard-type reactions in thermally processed foods. These food-derived CNDs share key properties—such as fluorescence and surface functionality—with engineered nanodots used in biomedical applications. However, no published studies to date demonstrate the use of Maillard-derived CNDs in drug delivery, and their biomedical relevance remains theoretical.

Further research is needed to isolate Maillard-derived CNDs in sufficient quantities, evaluate their safety, characterize their drug-loading capacities, and assess their biodistribution and cytotoxicity in vivo. A multidisciplinary approach will be essential to harness their functional potential while addressing associated health and safety concerns.

## Figures and Tables

**Table 1:** Flavor Compounds Identified in Maillard Reaction (MR).

Compound Type	Examples	Ref.
Acids	Butyric acid, isovaleric acid	[40]
Alcohols	1-hexanol, 2-phenylethanol	[34]
Aldehydes	Hexanal, nonanal, furfural	[35]
Carbonyl Compounds	Acetoin, diacetyl (2,3-butanedione)	[37]
Heterocyclic Compounds	Pyrazines, pyrroles, furans	[41]
Ketones	2-heptanone, 3-octanone, 2-pentanone	[36]
Sulfur Compounds	Dimethyl disulfide, methional	[38]

**Table 2:** Color Compounds Identified in Maillard Reaction (MR).

Compound Type	Examples	Ref.
Furan Derivatives	Furfural, 5-hydroxymethylfurfural (HMF)	[45]
Imidazoles	4(5)-methylimidazole	[46]
Nitrogenous Polymers	Melanoidins (high molecular weight, dark-brown pigments)	[47]
Phenolic Compounds	Phenol, hydroxyphenylacetaldehyde	[5]
Pyrazines	2-ethyl-3,5-dimethylpyrazine, methylpyrazine	[48]
Pyrroles	Pyrrole, substituted pyrroles	[49]
Reaction Products	Glucose–lysine browning products, caramel-like pigments	[50]

**Table 3:** Some evidence for the formation of carbon nanodots (CNDs) through the Maillard reaction (MR).

Food Matrix	Formation/Methods	Key Findings	Ref.
Bakery products	Baking (NaOH immersion step involved)	CNDs < 10 nm formed during baking; NaOH pretreatment facilitated CND formation and improved yield.	[54]
Coffee beans	Roasting process	Fluorescent CNDs identified; their presence correlated with caffeine content and roast intensity.	[52]
Milk	Synthesized via the hydrothermal method	The resulting CNDs demonstrated good sensitivity for detecting copper ions, with potential applications in food safety monitoring.	[57]
Mushroom powder	Pyrolysis of *Pleurotus ostreatus*	A strong positive correlation between the carbon/nitrogen ratio and CND yield; mushroom biomass is a viable precursor.	[51]
Spices	Pyrolysis of black pepper, turmeric, cysteine, clove, ginger, and chili spices	CNDs showed enhanced bioavailability, potent antioxidant activity, and improved biological functionality.	[58]
Starch-rich cooked foods	High-Temperature Processed Starch/Myristic Acid	Produced CNDs exhibited strong fluorescence; demonstrated potential for immunomodulation via cytokine regulation.	[59]

**Table 4:** The impact of Maillard reaction (MR) on food quality and sensory attributes.

Food Type	Key Findings	Impact	Ref.
Meat Products	A meaty flavor additive was developed using soybean meal hydrolysate and xylose via the Maillard reaction at 120 °C for 120 min with 10% cysteine. The product contained 4.941 μmol/mL of free amino acids and 50 volatile compounds, including mercaptans, sulfur-substituted furans, pyrazines, aldehydes, and esters.	High antioxidant activity; rich in volatile flavor compounds; potential as a food additive	[72]
Baked Goods	MR during baking leads to the formation of color and flavor compounds and potentially toxic substances like AGEs and HMF. Ingredients like butter, sugar, and eggs influence MR extent and sensory quality.	Flavor and color formation; risk of toxic MRPs	[73]
Dairy Products	Non-enzymatic browning and MR contribute to caramel and roasted flavors in milk powders but can also result in off-flavors and sedimentation. Browning issues in skim milk powders can lead to consumer complaints.	Both desirable and undesirable effects: flavor, off-odors, browning	[74]
Vegetables	MR in processed vegetables can enhance flavor but also produce toxic compounds. Reactions involve proteins, polysaccharides, and polyamines, especially during storage and thermal processing.	Flavor enhancement: potential health risks	[71]
Fruits	While MR can improve the sensory quality of fruit-based products, it can also lead to the formation of toxic Heterocyclic Aromatic Amines (HAAs). Advances suggest MRs can occur without heat, through green processing methods, challenging traditional assumptions.	Sensory improvement and potential toxicity also occur in non-thermal processes.	[75]

**Table 5:** Key Findings on Carbon Nanodots for Drug Delivery Applications.

Shynthesis Method	Optical Properties	Toxicity	Drug Delivery Potential	Key Findings	Ref.
Dry and solution-based techniques	Size- and wavelength-dependent luminescence; resistant to photobleaching; non-blinking	Generally non-toxic, but certain forms may pose risks	Highlights potential use but notes need for further testing	Introduced foundational knowledge of C-dot fluorescence and synthesis; raised awareness of potential health concerns tied to specific structures	[99]
Sugar-derived C-dots in various solvents	Emission is strongly influenced by the solvent environment; tunable fluorescence	Low toxicity; highlights the need for safety assessments	Supports application in bioimaging and drug delivery	Demonstrated how structural and solvent variables influence C-dot behavior; encouraged deeper study of formation and emission mechanisms for food and drug safety	[100]
Various methods, with focus on functionalization	Fluorescent emission is useful for imaging and therapeutic tracking	Emphasizes the low cytotoxicity of CQDs	Strong drug loading and release capabilities via covalent bonding	Highlighted CQDs’ promise in multifunctional roles, including simultaneous imaging and drug release; discussed controllable delivery methods	[101]
One-pot hydrothermal synthesis	Strong fluorescence with cell-type specificity; stable in aqueous media	Low cytotoxicity confirmed in cancer cells.	Effective for imaging and drug tracing.	Nitrogen-doped CNDs have been shown to differentiate cancer cells with low toxicity and high water stability	[102]
Review of multiple synthesis approaches	Describes diverse fluorescence behaviors for targeting and imaging	Calls for detailed toxicological evaluation	Responsive to pH/temperature triggers as nanocarriers	Summarized nano-carbon drug carriers; emphasized stimulus-responsiveness and rigorous safety evaluation required	[103]

## Data Availability

Not applicable.
